# Green Fabrication of Freestanding Piezoceramic Films for Energy Harvesting and Virus Detection

**DOI:** 10.1007/s40820-023-01105-6

**Published:** 2023-05-20

**Authors:** Shiyuan Liu, Junchen Liao, Xin Huang, Zhuomin Zhang, Weijun Wang, Xuyang Wang, Yao Shan, Pengyu Li, Ying Hong, Zehua Peng, Xuemu Li, Bee Luan Khoo, Johnny C. Ho, Zhengbao Yang

**Affiliations:** 1 Department of Mechanical and Aerospace Engineering, Hong Kong University of Science and Technology, Clear Water Bay, Hong Kong, People’s Republic of China; 2 Department of Mechanical Engineering, City University of Hong Kong, Hong Kong, Hong Kong SAR PRC; 3 Department of Biomedical Engineering, City University of Hong Kong, Hong Kong, Hong Kong SAR PRC; 4 Department of Materials Science and Engineering, City University of Hong Kong, Hong Kong, Hong Kong SAR PRC; 5 Department of Materials Science and Engineering, State Key Laboratory of Terahertz and Millimeter Waves, City University of Hong Kong, Kowloon, Hong Kong SAR PRC; 6 Guangdong Provincial Key Laboratory of Functional Oxide Materials and Devices, Southern University of Science and Technology, Shenzhen, 518055 Guangdong People’s Republic of China

**Keywords:** Van der Waals, Water stripping, Freestanding oxide films, Energy harvesting, Virus sensor

## Abstract

Most electronics such as sensors, actuators and energy harvesters need piezoceramic films to interconvert mechanical and electrical energy. Transferring the ceramic films from their growth substrates for assembling electronic devices commonly requires chemical or physical etching, which comes at the sacrifice of the substrate materials, film cracks, and environmental contamination. Here, we introduce a van der Waals stripping method to fabricate large-area and freestanding piezoceramic thin films in a simple, green, and cost-effective manner. The introduction of the quasi van der Waals epitaxial platinum layer enables the capillary force of water to drive the separation process of the film and substrate interface. The fabricated lead-free film, ${\text{Ba}}_{{{0}{\text{.85}}}} {\text{Ca}}_{{{0}{\text{.15}}}} {\text{Zr}}_{{{0}{\text{.1}}}} {\text{Ti}}_{{{0}{\text{.9}}}} {\text{O}}_{{3}}$ (BCZT), shows a high piezoelectric coefficient *d*_33_ = 209 ± 10 pm V^−1^ and outstanding flexibility of maximum strain 2%. The freestanding feature enables a wide application scenario, including micro energy harvesting, and covid-19 spike protein detection. We further conduct a life cycle analysis and quantify the low energy consumption and low pollution of the water-based stripping film method.

**Supplementary Information:**

The online version contains supplementary material available at 10.1007/s40820-023-01105-6.

## Highlights

- Freestanding lead-free piezoceramic thin films can be derived via water peeling.
- The* d*_33_ value of the freestanding BCZT thin film reaches 209 pm V^−1^.
- The freestanding piezoceramic films obtain versatile application scenarios such as energy harvest and virus detection.

**Supplementary Information:**

The online version contains supplementary material available at 10.1007/s40820-023-01105-6.

## Graphical abstract

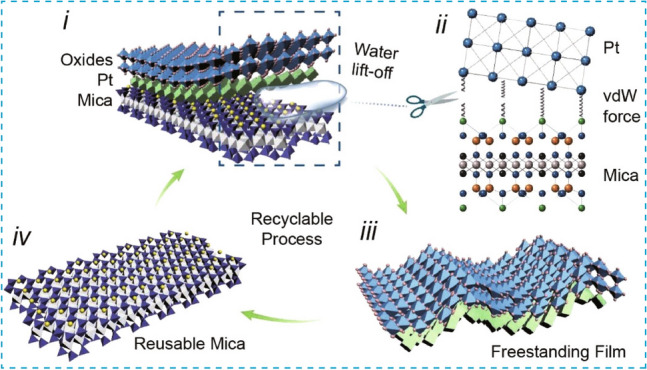

## Introduction

Piezoelectric films are essential in modern electronic devices as energy conversion units [1–4]. Existing technologies require rigid and high-temperature resistant substrates to grow piezoceramic films, followed by external processes to transfer the films to target surfaces, such as laser lift-off, dry etching, chemical etching, or buffer layer dissolution [5–9]. Since these transfer methods focus on breaking the interface at the bottom surface of the film, the intense stimuli, such as thermal stress and chemical destructions, inevitably cause residual stress and defects to films [10–12]. Furthermore, etching undoubtedly means the disposable use of the substrate material, which accompanies the vast energy consumption and the emission of strong corrosive chemicals that significantly damage the already vulnerable environment. Realizing the reuse of substrates requires the non-destructive separation of the film and the substrate, while it is almost impossible for piezoceramic films due to the growth interface dominated by covalent bonds [13].

We are inspired by a classic demonstration of peeling hydrophobic drawings of the dry-erase marker with water (Fig. S1). This facile peeling process enables a tailorable preparation and storage of inherently soft thin-film materials, such as polyimide and polydimethylsiloxane (PDMS). Whether can such a water-based peeling method work with brittle piezoceramics? It is possible if we consider the recent reports [8, 14–16], where metal-oxide films with a thickness reduced down to nanometers become much more flexible than their bulk counterparts.

Here, we report a van der Waals (vdW) stripping method to fabricate large-area and lead-free piezoceramic films using natural water. Mica, an emerging 2D layered structural material, is utilized as the thin-film growth substrate [17–19]. The success growth of PZT thin films on mica sheets has created a new era for flexible piezoceramic films [20–22]. It has been manifested that the heteroepitaxial growth through the physical deposition of 3D materials onto 2D substrates is a kind of vdW epitaxy, that is, the interface of the two materials is weak vdW interactions [23, 24]. Based on this, we can deposit a thin metal layer onto a mica substrate to form a vdW heterostructure. In the meantime, the metal film also serves as a buffer layer and a bottom electrode functional layer of the next-grown piezoceramic film. Within such a heterostructure, the interface between the film and the substrate can be separated by the capillary force of water. Considering the toxicity of lead to living organisms and the prohibition of lead-containing devices by Waste Electrical and Electronic Equipment (WEEE) and Restriction of Hazardous Substrates (RoHS), in this work, we select BCZT, a lead-free piezoceramics with good potential to substitute the lead-contained material [25], to prepare the freestanding lead-free BCZT thin films with a large area (2 cm × 2 cm), high-performance (*d*_33_ = 209 ± 10 pm V^−1^). Thanks to the freestanding feature, the derived films are totally released from the substrates and can be easily integrated with electrodes and target substrates to assemble flexible energy harvesters and biosensors. Finally, we conduct a life cycle assessment to evaluate the environmental impacts of the vdW stripping method, showing its advantages of low cost and low power consumption.

## Experimental Section

### Growth of BCZT Thin Films

The purchased mica substrates (Taiyuan Fluorophlogopite Mica Company Ltd.) are cleaned with acetone, ethanol, and deionized water before the experiments. A thin layer of Pt film (~ 10 nm) is deposited onto the mica surface via magnetron sputtering (Q150TS, QuorumTech) as the van der Waals epitaxial growth layer. The precursor of BCZT sol includes barium acetate, calcium acetate, titanium isopropoxide, and zirconium n-propoxide. The preparation details have been listed in our previous research [14]. The concentration of the sol used in this work is 0.2 M. The BCZT sol is dropped onto the Pt-coated mica surface and spun at 3000 rpm for 30 s, followed by volatilizing the organic matter at 400 °C, 10 min, and sintered at 800 °C, 20 min. After repeating the process twice, a BCZT thin film with 400 nm thickness is successfully prepared.

### Transfer of BCZT Thin Films

The ethylene–vinyl acetate (EVA, 4% wt of ethylene–vinyl) is firstly dissolved in toluene with 0.1 g mL^−1^. Then the EVA solution is spin-coated onto the BCZT surface with 1000 rpm for 30 s as the supporting layer, followed by drying at 140 °C for 10 min. When exposing the cross section of EVA/BCZT/Pt/Mica sample to the side of the water droplet, the water will peel off the EVA/BCZT/Pt thin film from the mica by the capillary force. The film will float onto the water surface. When transferring the film onto any substrates, the target substrate needs to dip into the water and pull up the thin film carefully to maintain the integrity of the material after drying in the air. Finally, the supporting layer EVA can be dissolved by toluene droplets and reused the next time.

### Materials Characterizations

All the SEM and EDS results are derived from the microscope (FEI, Quanta 450) integrated with the instrument (INCA Energy 200). The contact angles are measured by the goniometer (OCA25, Dataphysics). The crystallinity is characterized by the XRD (Rigaku Smartlab) and the Raman spectrometer (Perkinelmer Raman station 400F). The ferroelectricity is conducted on an Asylum Research MFP-3D Infinity AFM.

### Cell Viability Experiments

The myofibroblast cell line WPMY-1 is purchased from American Type Culture Collection (ATCC). The cultured medium is Dulbecco’s modified Eagle’s medium (DMEM, Gibco, #11875085, USA) supplemented with 5% fetal bovine serum (FBS, Gibco, #10270106, USA) and 1% penicillin–streptomycin (Gibco, #15140122, USA). Cells are cultured under 5% CO_2_ humidified atmosphere at 37 °C. Media are changed every 2 days and cells are passaged at 80% confluence.

BCZT, PDMS encapsulated BCZT, and PDMS membrane samples are placed in a 24-well plate. Samples were disinfected by 75% overnight and were exposed under UV-light for sterilization. Before cell seeding, samples were washed by PBS to remove the ethanol residues. The myofibroblast is seeded into the 24 well plate at a density of 1.2 × 10^4^ mL^−1^. Fresh medium were changed every two days. After 1, 3 and 5 days of incubation, the cells are stained with 5 µM Calcein AM (Invitrogen, #C3100MP, USA) under 37 ℃ for 20 min and 5 µM Propidium Iodide (PI) (Sigma-Aldrich, #81,845, USA) at room temperature for 1 min to identify the live and dead cells. The stained samples are imaged with a fluorescence microscope (Nikon, Eclipse Ci-L, Japan).

All fluorescent images are processed by Image J software (National Institutes of Health, USA) and automated algorithms are generated to count both the cell numbers and fluorescent intensity from the derived images. The results are expressed as means ± standard deviation of three independent trials. One-way ANOVA and Student’s *t*-test are conducted to evaluate associations between independent variables, and the statistical significance is defined as *p < 0.01.

### Piezoelectric Performances Characterization

The derived freestanding BCZT thin film is placed on a flat stainless steel disk with a diameter of 1 cm. Before measuring the piezoelectric response, the film is firstly polarized under a corona electric field under 10 kV cm^−1^ at room temperature and stored in the ambient environment for two days to eliminate the electrostatic interference. The PFM test is conducted by the atomic force microscope (Asylum Cypher ES) under DARTSSPFM testing mode with the conductive tips (Arrow-EFM-10, Spring constant 1.6 N m^−1^). The contact mode is used and the piezoelectric response is obtained under the resonance frequency (331–339 kHz). The influence of the contact resonance can be eliminated by calculating the quality factor of the unit pixel.

To fabricate the FBEH, we coated two PDMS membranes of specified thicknesses onto glass plates. The Pt electrode layers are deposited onto the PDMS membranes. The freestanding BCZT thin film is encapsulated by the plasma-cleaned PDMS membranes. The open-circuit voltage output is recorded by a digital oscilloscope (Rohde & Schwarz RTE1024), and the short-circuit current is measured by a current preamplifier (Stanford Research SR570).

### Biosensor Experiments

The EVA/BCZT/Pt thin film is firstly transferred to a 20 nm thick Pt coated PDMS membrane with 200 μm. The EVA is removed by a small amount of toluene. A 2 mm diameter, 20 nm thick gold film is sputtered onto another 20 μm thick PDMS membrane to increase the signal strength and the gold deposited side is attached to the BCZT film for encapsulation. This device is clamped and fixed by two $3 \;{\text{cm}}\; \times \; 4\;{\text{cm}}$, 1 mm thick PCBs, with the upper and lower electrodes each directly touching the board conductor and integrated into a BNC connector. The resonant frequency is characterized by the ENA Network Analyzer (E5061B, Keysight).

Before starting the experiment, the PDMS surface needs to be grafted with carboxyl groups. Firstly, PDMS is immersed in benzophenone (10 wt% in water, TCI, > 99%) for 4 h, dried for 5 h thick with UV light for 5 min and soaked with ethanol. After drying, the surface is dripped with 50% aqueous acrylic solution and irradiated with UV light for 5 min. After drying, the surface is dripped with 50% aqueous acrylic solution and irradiated with UV light for 5 min, and the surface is cleaned with deionized water. The grafted results are characterized by FTIR (Tensor 2, Bruker). The antibodies with fluorescent labeling (SARS-CoV-2 Spike RBD HL1002) are purchased from GeneTax. The samples are imaged with a fluorescence microscope (Nikon, Eclipse Ci-L, Japan).

## Results and Discussion

### Capillary Stripping of Piezoceramic Thin Films

Peeling is a classical scientific problem. As systematically explained by Griffith and Kendall, the process of peeling is the quasistatic propagation of the interfacial crack between the film and the substrate [26–29]. Based on this, most research separates thin films from their host substrates by optimizing the interfacial surface energy and the mechanical properties of thin films, which brings the dawn for the defect-free transfer of 2D materials and the construction of 3D buckling structures [30–32]. Among them, taking advantage of the capillary effect of water as the driving force of peeling is an emerging method to achieve non-destructive thin film exfoliation [33, 34]. Capillary force originates from the intermolecular forces and closely relies on the surface tension and adhesive forces between the liquid and the substrate. When the interaction force between the film and the substrate is small enough, the capillary force of water will produce a crack at the interface and keep propagating until the film is totally detached from the substrate [29]. To better understand the phenomenon, we use a commercial dry-erase marker to write “CityU” on a mica substrate. The pattern can be easily stripped from mica and floated onto the water surface, as shown in Fig. S1a. The schematic Fig. S1b describes capillary peeling and the ceramic thin film transfer is similar to the process. The relation *G* = *W* is satisfied when the crack just occurs, where *G* is the effective mechanical energy applied to separate the two materials; *W* is the thermodynamic work of adhesion that is determined by the interfacial energy of each interface in this system, as illustrated in Fig. S1c. The adhesion work W can be expressed as Eq. 1:

1 $$W = \gamma_{{{\text{SL}}}} + \gamma_{{{\text{LF}}}} - \gamma_{{{\text{SF}}}} ,$$

where $\gamma_{{{\text{SL}}}} , \gamma_{{{\text{LF}}}} , \gamma_{{{\text{LF}}}}$ are the interfacial energy of the substrate with the liquid, the liquid with the film, and the substrate with the film, respectively. According to Young’s Dupre equation, the relation of each interfacial energy is:

2 $$\gamma_{{{\text{LF}}}} \cos \theta = \gamma_{{{\text{SL}}}} - \gamma_{{{\text{SF}}}} .$$

Then the adhesion work can be expressed as:

3 $$W = \gamma_{{{\text{LF}}}} \left( {1 - \cos \theta } \right),$$

where $\theta$ is the contact angle of the liquid on the substrate [26–29]. To improve the success rate of the film peeling process, we must reduce the magnitude of the thermodynamic work *W* as much as possible. According to Eq. 3, a more hydrophilic substrate and a more hydrophobic thin film are necessary for the interface separation.

Mica is an ideal substrate material due to its highly hydrophilic and high-temperature resistance, as shown in Fig. S2a. However, for most ceramic materials, especially the BCZT thin film, the contact angle is inherently small unless preparing complicated micro-patterns onto the surface, which will significantly increase the fabrication cost.

To address this issue, we introduce a top surface hydrophobic polymer layer and a bottom metal buffer layer into the system. Before growing the BCZT film, a thin layer of Pt film (~ 10 nm) is firstly deposited on the mica substrate via magnetron sputtering. The van der Waals epitaxial-grown Pt film has a weak interaction with the substrate that can easily be peeled off by external forces. Furthermore, Pt is not that hydrophilic with a 40° contact angle, where the interfacial energy is much lower than that of BCZT thin film (3°). For the top surface layer, we use non-toxic thermoplastic ethylene–vinyl acetate (EVA). The 87°contact angle (Fig. S2d) shows its good potential to reduce the force required for separating the interface. Besides, the high flexibility and good mechanical strength of the top EVA layer ensure the integrity of the ceramic film during the transfer process. Using these preparations, we assemble the Janus-like stacked films and peel them off via the capillary force.

**Fig. 1:**
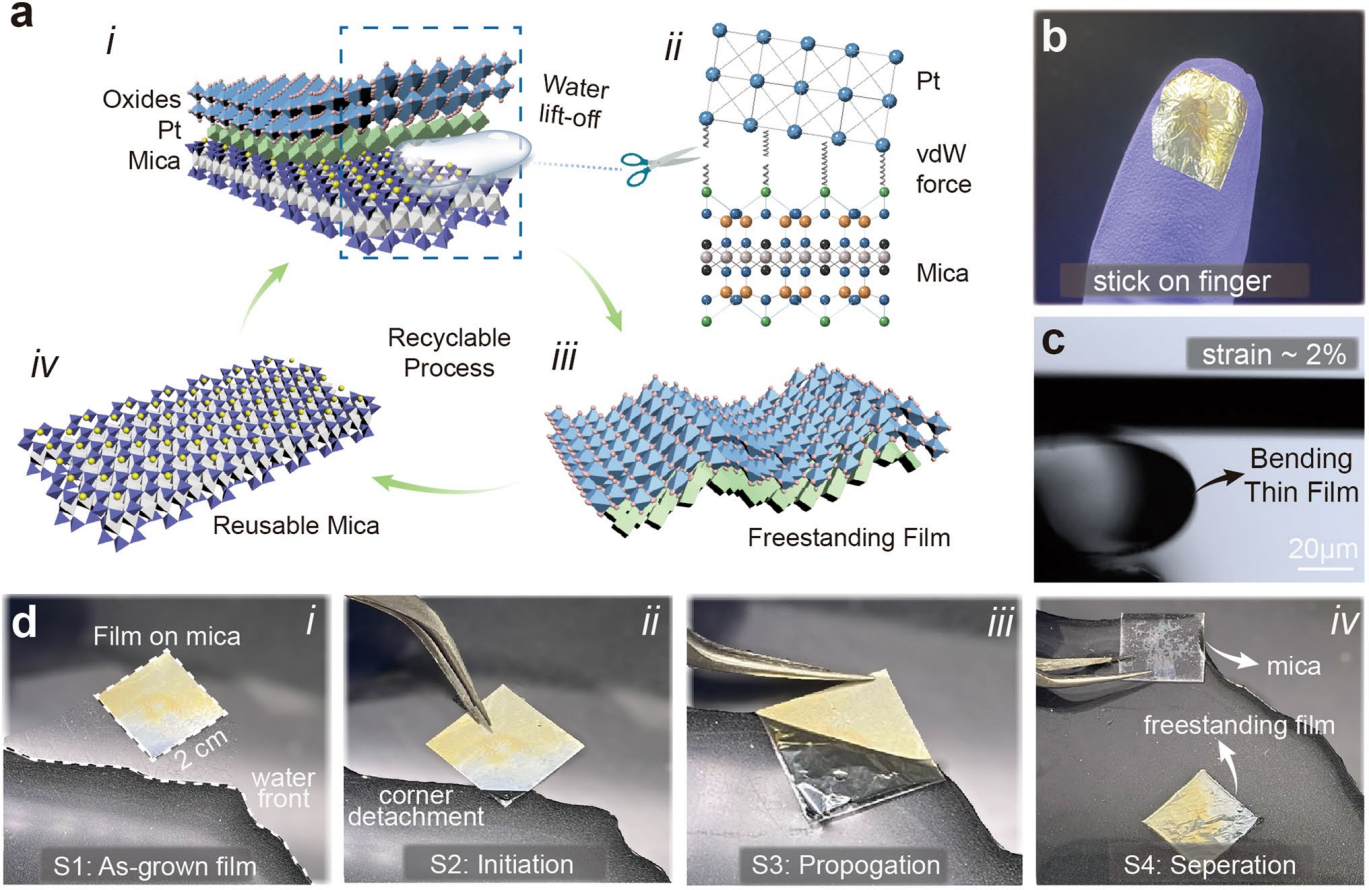
Illustration of the van der Waals stripping of piezoceramic thin films. **a** Schematics of the van der Waals lift-off process: (i) the waterfront opens the interface between mica and piezoceramic/Pt layer. (ii) on the microscopic view, Pt layer and mica sheets are bonded via weak van der Waals forces and can be easily cut off by an external force, such as the water in this work. (iii) the freestanding thin film is derived when the water totally penetrates the interface. (iv) the mica can be recycled due to the non-destructive process. **b** Freestanding BCZT thin film with supporting layer EVA and bottom electrode Pt stands on the finger with gloves. **c** Bending behavior of the freestanding BCZT film piece under an external force. **d** Photographs of the thin film transfer process. (i) the as-grown BCZT thin film on Pt/mica coated by an EVA layer with 2 cm × 2 cm. (ii) the water lift up a small conner of the film. (iii) the water keeps penetrating the interface. (iv) the freestanding film floats on the water surface

Figure 1a shows the capillary transfer process. The capillary force of water first creates a crack between the Pt and mica interface, and then the crack keeps propagating throughout the interface until the whole film is lifted off. Here, as illustrated in Fig. 1a(ii), the capillary force serves as a scissor to cut off the van der Waals “springs” between the mica substrate and the Pt layer. Since there are no destructions on mica sheets, the mica substrates can be reused in the next round of thin film coating. The photograph of the process of water peeling of the film is shown in Fig. 1d. The prepared film sample is first placed beside the water surface. When the film slowly moves closer to the liquid surface, the capillary force will gently uncover one corner of the film, as illustrated in Fig. 1d(ii). As shown in Fig. 1d(iii), the waterfront will gradually penetrate through the films interface, and the process of the interfacial separation can be assisted by increasing the water fluctuation. Finally, the freestanding film floats on the water surface and the mica sheets submerge in the water, as shown in Fig. 1d(iv). Here, water also acts as a flowing, deformation-free substrate that effectively helps to release residual stresses in the film and avoid deformation during the transfer process. Since the ceramic film is inherently brittle, the top EVA layer presents as a supporting layer to prevent any potential fracture caused by external stimulus on a large piece of the freestanding film. Then the film can then be placed on curved surfaces after lifting from the water, such as the finger and water bubbles (Figs. 1b and S3).

After transferring the film onto target substrates, the supporting EVA layer can be removed by dissolving it in organic solvents. Notably that the removal process has to be carefully controlled since quick dissolution may cause damage to the ceramic films. To avoid the possible destructions, the sample should be firstly fixed on the target substrate, and following by adding a small amount of toluene to dissolve the EVA layer. The toluene is a kind of volatile solvent and has no chemical reaction with ceramic films, thus it will not remain on the sample or pollute the BCZT film in this process. The freestanding BCZT film (~ 400 nm) can withstand large bending deformations, which is observed by an optical microscope, as demonstrated in Video S1. One end of a small BCZT film piece with 110 μm length and 50 μm width is fixed to form a cantilever beam. The ceramic film is bent by applying external stress to the free end. As shown in Fig. 1c, the film reaches a maximum bending strain of about 2% (for calculations, see supplementary), and quickly recovers to the initial state when withdrawing the external force. Notably that for bulky ceramic materials, the fracture strain is 0.2–0.4%. Our fabricated freestanding 0.4 μm-thick films show 5–10 times higher bending strength than bulky ceramics [35, 36].

### Fundamental Characterization

**Fig. 2:**
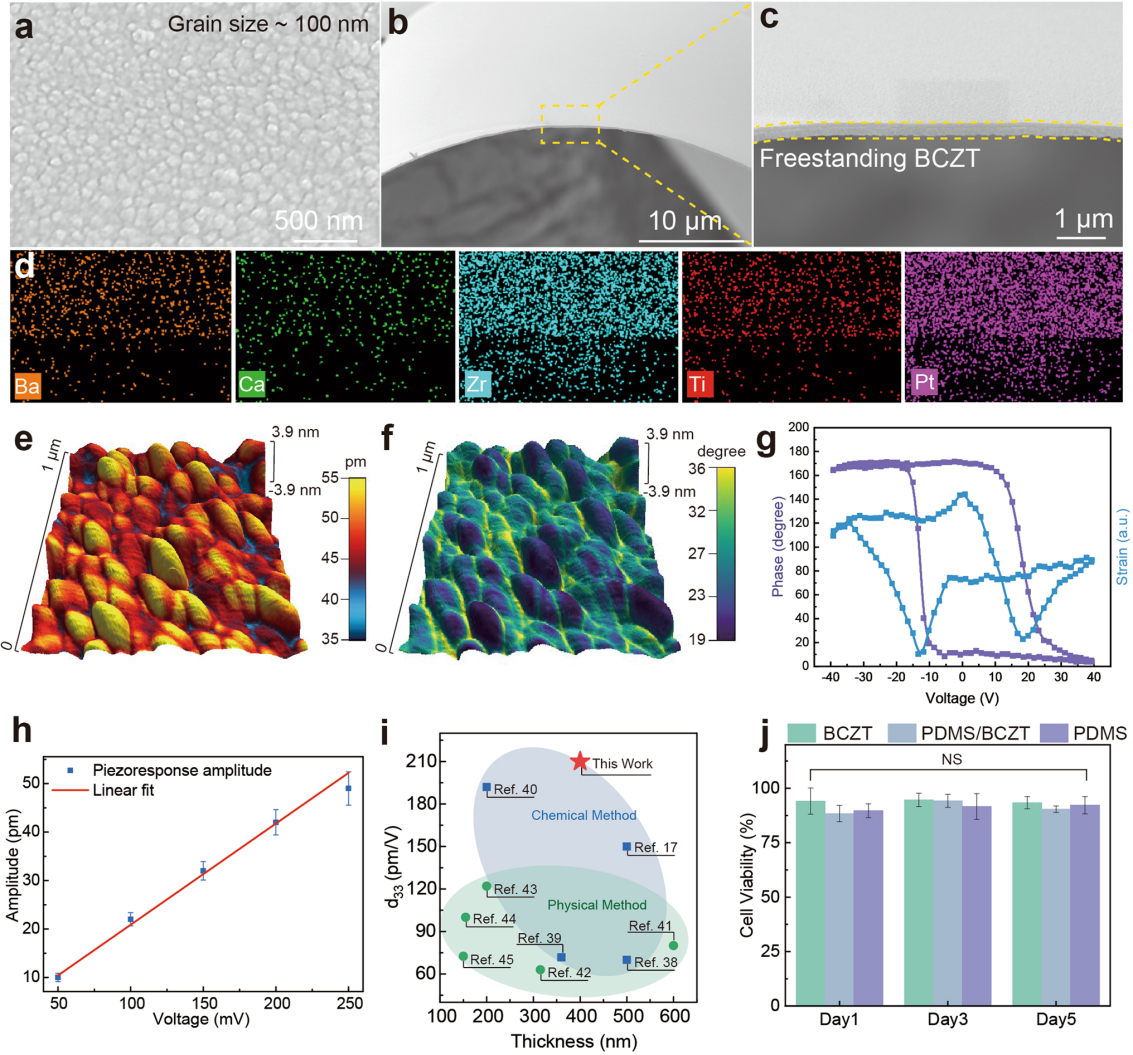
Characterizations of the freestanding BCZT thin film. **a** SEM image of the top surface of the BCZT thin film. **b** SEM image of the cross sectional view of a piece of freestanding BCZT thin film and **c** the magnified image. **d** EDS results. **e** PFM piezoelectric response amplitude under 200 mV driven voltage. **f** Corresponding phase distribution. **g** Phase-voltage hysteresis loop and the strain-voltage hysteresis loop. **h** Linear fitting of the piezoelectric response under 50 to 250 mV driven voltages. **i** Thickness vs *d*_33_ comparison of BCZT thin film in recent years, classified by physical and chemical preparation methods. **j** Cell viability of myofibroblasts cultured on the surface of BCZT film, the BCZT film encapsulated by a 10 μm-thick PDMS layer, and a pure PDMS membrane after 1, 3, and 5 day incubation. *NS* no significance

The characteristics of the BCZT thin film are investigated. The scanning electron microscopy (SEM) top surface view of the BCZT film initially grown on Pt/mica substrate is shown in Fig. 2a, and the cross-sectional view is shown in Fig. S5a. The freestanding piezoceramic film exhibits a smooth, crack-free, and highly densified ceramic film composed of grains of approximately 100 nm. The cross-sectional view of a large-area freestanding BCZT/Pt thin film with about 400 nm on the EVA supporting layer is shown in Fig. S5b. The film after removing the EVA layer is shown in Fig. 2b, and the zoom-in image is shown in Fig. 2c. The element composition of the film is manifested by electron dispersion spectroscopy, as shown in Fig. 2d. There are barely any cracks or pores shown on the film, verifying the water-peeling process will not bring severe damage to the material supported by the EVA layer.

Taking advantage of the non-destructive nature of the water transfer process, we reuse the mica sheets as growth substrates. We use X-ray diffraction (XRD) and Raman spectroscopy to characterize the crystallinity of the BCZT thin films multiplied grown on a single mica substrate. Besides the mica substrate, the BCZT film grown on mica and transferred for the first and second time is examined, as shown in Fig. S6. All types of BCZT films show the typical perovskite phase. Affected by the mica substrate, the films on mica substrates show the typical signal of the mica crystal at 2$\theta = 26.8^\circ$.

In contrast, the corresponding peaks are disappeared on the transferred films manifesting films are totally lifted off from mica substrates. It is obvious that the XRD spectrum of the BCZT films does not change significantly regardless of whether the mica is multiple used, or films are transferred. This proves that the water-peeling process will not affect the crystallinity of the films, and the reuse of mica substrates is also feasible. The scattering bands in the Raman spectrum correspond to the tetragonal phase in the perovskite structure [37], as shown in Fig. S7.

The piezoelectric properties of the freestanding BCZT thin film are quantitatively investigated by a piezoresponse force microscope (PFM). In the resonance frequency (331–339 kHz) mode, we measure the piezoelectric displacement response of the film under 200 mV stimulation within an area (1 μm × 1 μm), as shown in Fig. 2e-f. After deducting the effect of excessive displacement caused by the resonance, the detected displacement amplitude reaches 35–55 pm. The uniform distribution of the phase angle indicates effective polarization. The ferroelectricity of the freestanding film is also examined as shown in Fig. 2g, where the phase-voltage and strain-voltage hysteresis loops show the application potential in ferroelectric devices. To verify the accuracy of the piezoelectric response, we apply the driven voltages ranging from 50 to 250 mV to detect the response amplitude. As shown in Fig. 2h, the piezoelectric response of the BCZT film shows a linear relationship between the increasingly driving voltage. *d*_33_ = 209 ± 10 pm V^−1^ is calculated by the slope of the straight line linearly fitting from the data. We compare the recent works that report the ${\text{d}}_{33}$ value and the thickness of BCZT thin film in Fig. 2i, further exhibiting the application potential of the lead-free piezoelectric materials [38–45].

Biocompatibility is critical for biomedical applications. Although the toxicity of the BCZT ceramic is much lower than that of lead-containing piezoelectric materials, the biocompatibility of the BCZT films and the devices still needs to be examined for better use in living organisms. Herein, we compared the proliferation of myofibroblasts WPMY-1 on three materials: a BCZT film, the BCZT film encapsulated by a 10 μm-thick PDMS layer, and a pure PDMS membrane. The myofibroblasts are seeded on the surface of the three disinfected samples with a cell density of 1.2 × 10^4^ per sample. After incubating for 1, 3 and 5 days, the cell viability in each sample is evaluated. Due to the inherent hydrophilic properties of the BCZT film, as shown in Fig. S8, it is observed that myofibroblasts have an even distribution on the surface of the BCZT film after 1 day of incubation. They are more uniform after the following days’ incubation than myofibroblasts' morphology on the PDMS or PDMS encapsulated film. The BCZT film also displays good cell proliferation after five days of incubation, demonstrating that the reported BCZT material has no cytotoxicity and is applicable for skin-attachable applications. The BCZT film also shows high cell viability above 90% in all three points (Day 1: 94.11 $\pm$ 5.97%, Day 2: 94.60 $\pm$ 3.12%, Day 3: 93.37 $\pm$ 2.81%), which is comparable to the other samples as illustrated in Fig. 2j.

### Piezoelectric Performance

**Fig. 3:**
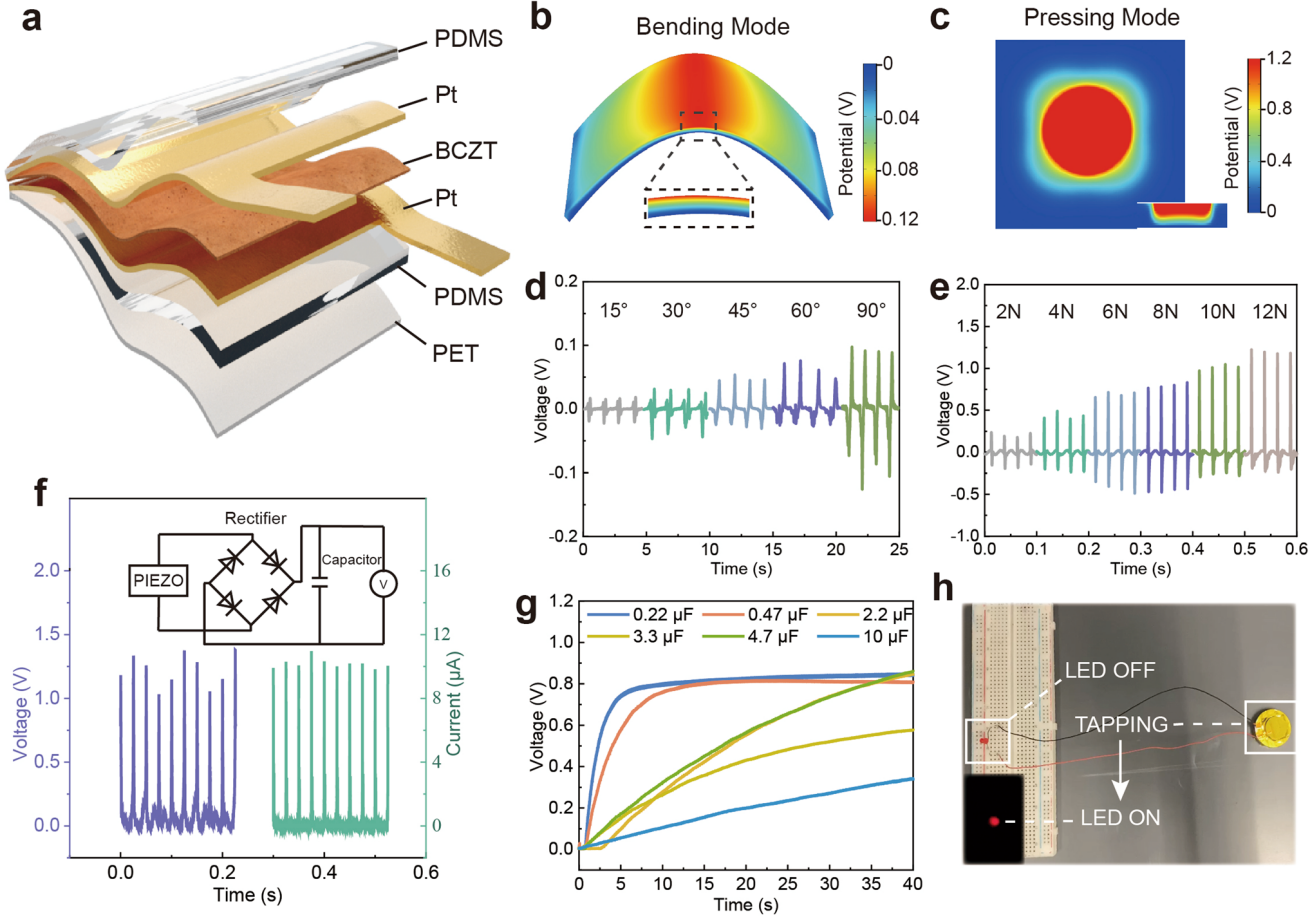
Piezoelectric performance of the freestanding BCZT thin film as an energy harvester. **a** Structure of the lead-free energy harvester based on 400 nm thick BCZT thin film. **b** Electrical potential distribution under bending estimiated by a finite element analysis. **c** Electrical potential distribution under compression estimiated by a finite element analysis **d** Voltage output of the energy harvester bent at different angles. **e** Voltage output of the FBEH under different pressing forces. **f** Rectified voltage and current signals of the FBEH. **g** Capacitor charging results in 40 s. **h** LED lights up powered by tapping the FBEH

To examine the piezoelectric performance, we assembly the freestanding BCZT film (2 cm × 2 cm) into a pair of parallel Pt electrodes as a freestanding BCZT-based energy harvester (FBEH) and encapsulated by polydimethylsiloxane (PDMS), as shown in Fig. 3a. The thickness of the bottom PDMS layer is larger than the top PDMS layer to eliminate the influence of the neutral layer on the piezoelectric properties. A finite element analysis is performed to investigate the operation mechanism of the FBEH. In the case of bending, the upper surface is subjected to tensile stress in the lateral direction, and the lower surface is subjected to compressive stress. In the meantime, there is compressive stress along the longitudinal direction. Thus, a piezoelectric potential difference is generated between the upper and lower surfaces, as shown in Fig. 3b.

Similarly, the potential difference will also be generated under normal compressive stress, as shown in Fig. 3c. The numerical study of the induced voltage confirms the validity of the design of the energy harvester. Based on the simulation results, bending and pressing modes are carried out to measure the piezoelectric output, and the experimental setup is illustrated in Fig. S9. At a bending angle of 90°, the obtained output signals show opposite phases when forward and reverse connecting the FBEH to the oscilloscope, indicating the triboelectric effect can be neglected in this system, as illustrated in Fig. S10a. The voltage output of the FBEH linearly increases from 20 to 120 mV under a bending stimulus that grows from 15° to 90° at the same rate, as shown in Fig. 3d. The short-circuit current output under the same stimuli is shown in Fig. S10b. Under normal stress, the output of the FBEH shows a linear relationship with the increase of the pressing force. The FBEH maintains a piezoelectric output of 1.2 V, 15 μA under 12 N, 20,000 cycles of pressing stimulus, demonstrating the ability to be assembled as a piezoelectric sensor as shown in Figs. 3e and S10c. The charge transfer is also measured as shown in Fig. S11. Notably that the dissymmetry of the generated signals result from the inconsistency between the deformation of the sample during the hammer strike and its spontaneous rebound.

Using the fabricated freestanding BCZT film, we developed a skin sensor and a mechanical energy harvester. As shown in Fig. 3f, a full bridge consisting of four diodes is connected in parallel with the FBEH to convert the AC signals output by the piezoelectric effect into positive signals. As shown in Fig. 3g, capacitors of 0.22 and 0.47 μF reach 0.8 V within 10 s under cyclic pressing at 40 Hz (an optimized working parameter for the shaker in this experiment). When the FBEH is directly connected to a red LED bulb, a firm tap on the FBEH will light up the LED, as shown in Fig. 3h. Moreover, the flexible FBEH can be attached to any skin surface to measure the corresponding human motion signals. As shown in Fig. S12, the bending finger generates signals up to 40 nA, and the bending elbows generate up to 30 nA. We also measure the piezoelectric response of the arm muscles under tension and relaxation in Fig. S12c. When the fist is clenched, a signal of − 10 nA is detected, and when the fist is relaxed, it will generate a signal with the opposite phase.

### Virus Detection via Freestanding Piezoceramic Films

The covid-19 virus pandemic was the most severe public health event in recent years, exacerbating the need for virus identification and surveillance [42]. Existing medical diagnostics commonly use combined chain reaction (PCR) amplification and enzyme-linked immunoassay for viruses, both of which require the addition of biomarkers for identification and are highly sensitive. However, the long analysis time and complex operational procedures are not necessarily applicable to the early qualitative identification of viruses. To reduce the cost and difficulty of detection, researchers have developed piezoelectric micromechanical balance-based biosensors [46–48]. The basic principle of the piezoelectric biosensor is to detect the resonant frequency of the piezoelectric material that shifts with the loading of the mass. Here we construct a microbalance for covid-19 spike-in protein detection to demonstrate the potential of freestanding piezoelectric films for biosensors.

**Fig. 4:**
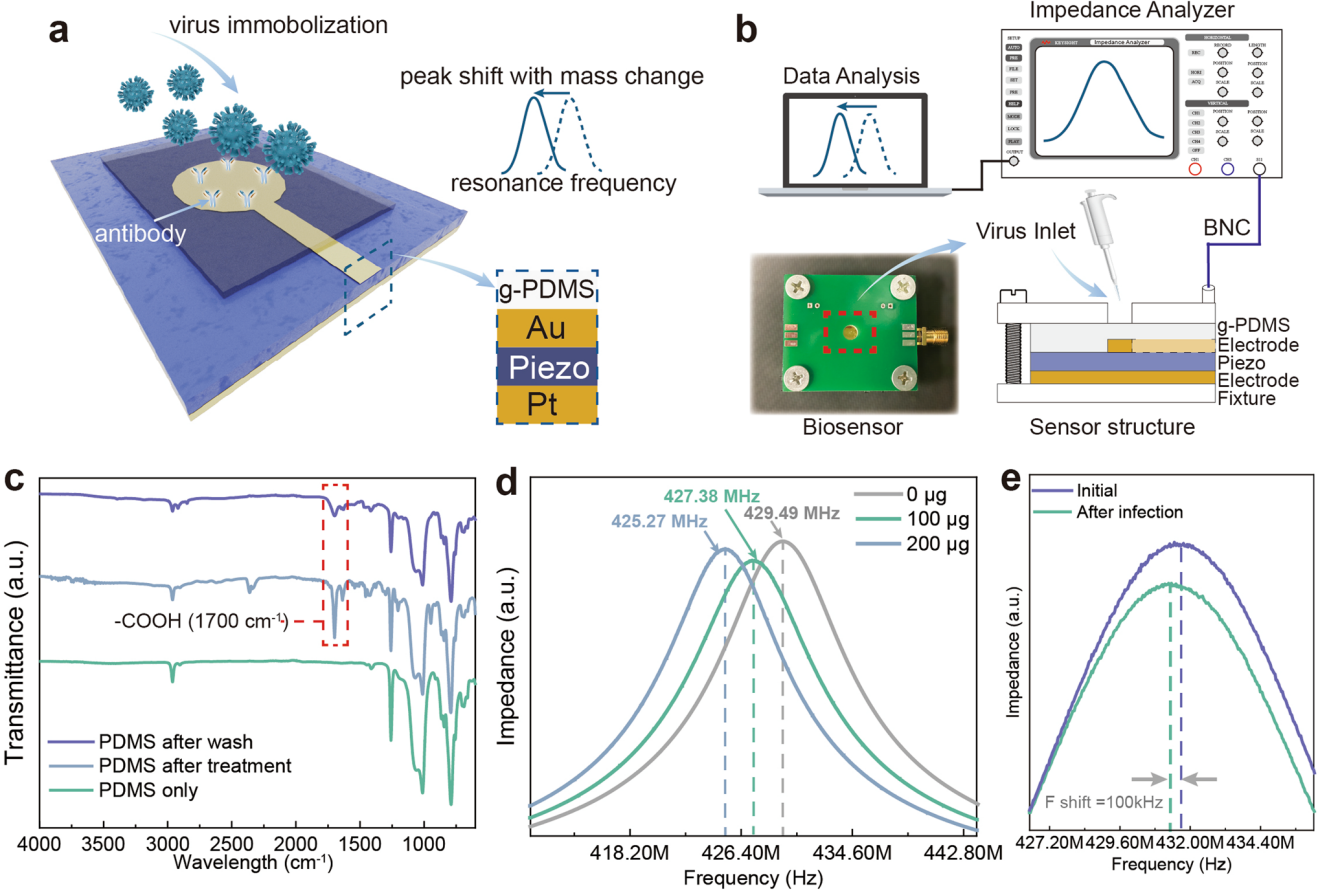
Virus biosensor. **a** Schematic of the piezoelectric thin film based biosensor. **b** Experiment setup of the virus detection and the structure of the biosensor. The biosensor is firstly fixed by two PCB plates with a BNC connector integrated. The sensor is connected to the impedance analyzer to detect the resonance spectrum in real time. The spectrum is recorded and analyzed on a computer. **c** FTIR results of the original PDMS, carboxyl grafted PDMS and the PDMS after water clean. **d** Calibration of mass change versus resonant frequency. **e** Result of the virus detection proves the existence of the covid-19 spike protein

As schematically illustrated in Fig. 4a, the biosensor consists of a freestanding BCZT thin film and two layers of metal electrodes on top and bottom. It is encapsulated with PDMS to ensure the film is free from damage in use. To detect the covid-19 spike-in protein, we immobilize the probe antibody on PDMS. Therefore, carboxyl grafting of PDMS is first required to facilitate the immobilization of antibody probes with amino groups [49, 50]. The detailed procedure of grafting is elaborated in the method section. Figure 4c shows the FTIR spectra comparison of the untreated and grafted PDMS. We find the carboxyl group peaks at 1700 cm^−1^ and retains after water washing. This means that the probe antibodies with fluorescent labeling are immobilized on the PDMS surface and are not removed by water rinsing (Fig. S13). To avoid any environmental interference, as illustrated in Fig. 4b, we fix the biosensor with a pair of fixtures with a BNC connector, and a small central hole is reserved for dropping samples. The accuracy of the biosensor is calibrated by dropping a quantitative amount of water drops. As shown in Fig. 4d, for each 10 µg drop of water, the resonant frequency is shifted by 2.11 MHz accordingly. After washing and drying the biosensor, a small amount of covid-19 spiked protein solution was added, left at room temperature for 10 min, and rinsed with deionized water. After drying, a shift in resonance frequency of about 100 kHz occurs, as shown in Fig. 4e, which means covid-19 presence. In addition to the covid-19 spike protein, the freestanding piezoceramic films-based sensor can identify any particles captured by the corresponding probes, indicating the great potential for market use.

### Life Ccycle Assessment of the van der Waals Stripping Process

Given that the use of capillary force to prepare freestanding ceramic films enables the recycling of the growth substrate and the absence of etching, there is potential to replace the existing thin film transfer process at a lower cost. We conduct a life cycle assessment to quantify the resource consumption of this process [51, 52]. Two main modeling techniques exist for LCA, including macro input–output analysis and process-based analysis. The former requires all activities of the study subject to be considered, while the latter involves selecting system boundaries for analysis. Due to the high similarity of the processes for preparing ceramic thin films by the sol–gel method, here we use the process-based analysis. We calculate the necessary parts of the van der Waals stripping process and compare it with a commonly used physical substrate removal process: deep reactive ion etching (DRIE) [7].

The goal of the LCA study is to estimate the potential life cycle impacts of the two substrate removal processes: vdW stripping and DRIE. The overall assessment follows the five steps: (i) obtaining the raw material requirements, the production, and the detailed processes of the two substrate removal technologies; (ii) analyzing the system, including establishing system boundaries, material composition, etc.; (iii) building the system inventory, including input, material flow, etc.; (iv) giving out the overall impact assessment and environmental profile evaluations; and (v) analyzing the technology and performance.

**Fig. 5:**
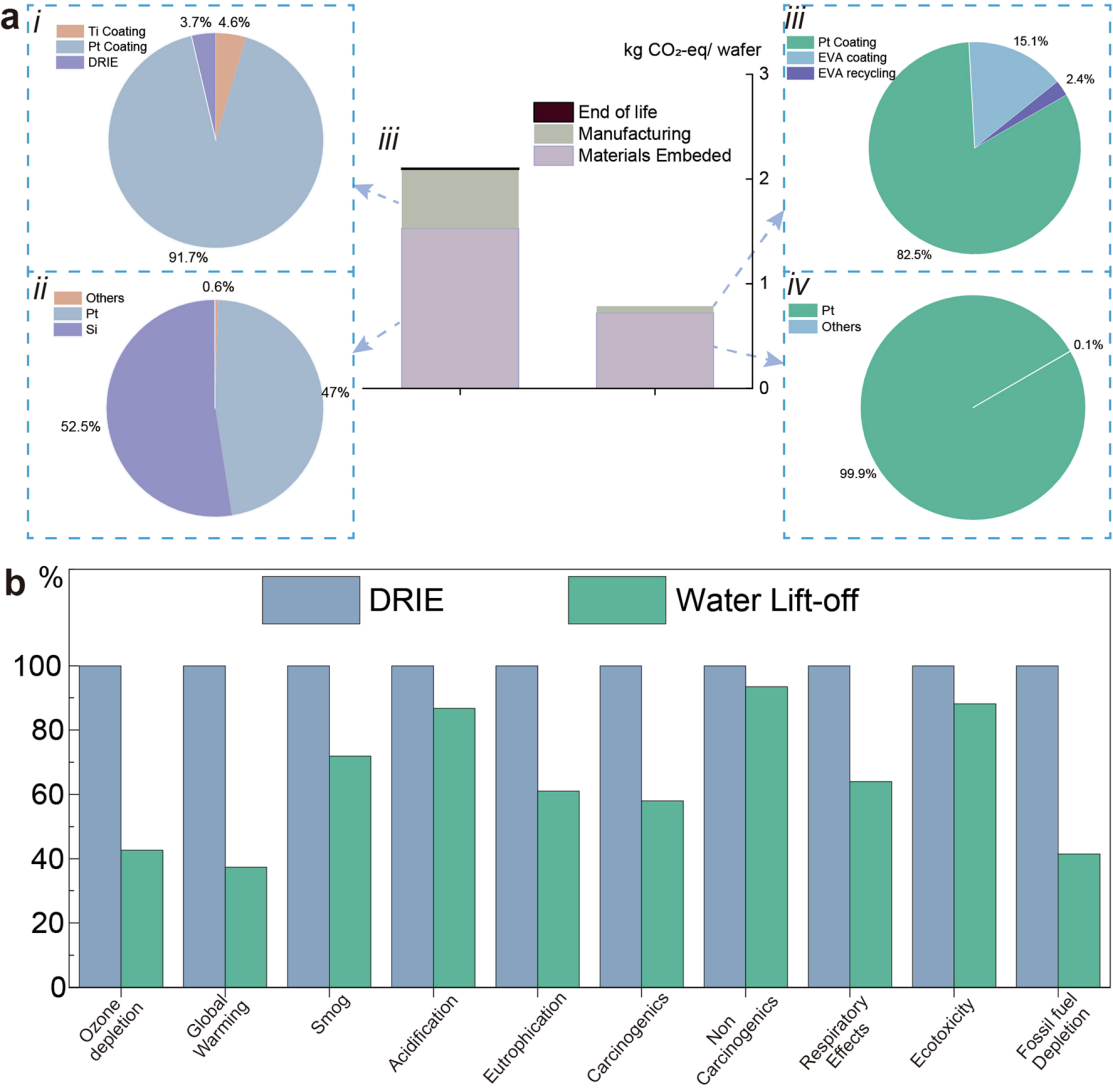
Life cycle analysis (LCA) results of the traditional physical DRIE etching method and the proposed van der Waals stripping method. **a** Contribution and comparison of the carbon footprint of the two processes. **b** Comparison per damage category, by summation of individual impacts, the higher impact set to equal 100%, using TRACI 2.1 V1.06/Canada 2005 methodology

The manufacturing route of van der Waals stripping and DRIE is shown in Fig. S14, and the raw materials requirements, and energy consumption are shown in Tables S1–S2. In this process, films are prepared by depositing on substrate materials and the substrates are removed by different processes to obtain the freestanding film. The system boundary and the lift cycle inventory are shown in Fig. S15. The functional unit is set as one wafer size with a 5.56 cm diameter. In this work, we focus on assessing the carbon footprint and the cumulative energy demand of both processes, as well as the life cycle impact of both in one functional unit. As shown in Fig. 5a, in both technologies, more than 90% of carbon emissions are generated by raw materials acquisition; DRIE generates 2.1 kg CO_2_-eq/wafer, while vdW stripping only emits 0.78 kg CO_2_-eq/wafer. The distribution of cumulative energy demand of the two technologies is also established, as shown in Fig. S16. It has similar patterns to the carbon footprint because the electricity consumption and carbon footprint are obtained by calculating some proper parameters in the evaluation process. The results show that DRIE requires about 32 MJ/wafer, while vdW stripping needs 12 MJ/wafer. Figure 5b shows the comparative LCA results. The environmental impact matric of DRIE is normalized to 100%, and it can be viewed that the vdW stripping process performs better than DRIE.

## Conclusions

The vdW stripping process has several important implications. First, this process provides substrate-free ceramic films that can be physically transferred to any expandable surface and integrated with electronic devices. Second, not limited to ceramic films, other materials, such as metals and polymers, can also be transferred by vdW process to obtain freestanding films. Finally, the process requires only water participation and the substrate mica can be recycled, significantly reducing production costs and operational complexity. The vdW stripping process breaks the limits that ceramic films can be fabricated only with silicon or other rigid substrates. The derived freestanding films endow great freedom to the design of piezoelectric electronics.

## Supplementary Information

Below is the link to the electronic supplementary material.

Supplementary file1 (PDF 1146 KB)

Supplementary file2 (MP4 5183 KB)
