# Supplementary material for: Green Fabrication of Freestanding Piezoceramic Films for Energy Harvesting and Virus Detection
Source: Nanomicro Lett. 2023 May 20;15:131. doi: 10.1007/s40820-023-01105-6 (PMC10199448; doi:10.1007/s40820-023-01105-6)
Supplement: Supplementary file 1 — Supplementary file1 (PDF 1146 KB) [file 40820_2023_1105_MOESM1_ESM.pdf]

Nano-Micro Letters

Supporting Information for

Green Fabrication of Freestanding Piezoceramic Films for Energy Harvesting and Virus Detection

Shiyuan Liu[1, 2, †], Junchen Liao[3, †], Xin Huang[1, 2, †], Zhuomin Zhang[1, 2, †], Weijun Wang[4], Xuyang Wang[6], Yao Shan[1,2], Pengyu Li[1, 2], Ying Hong[1, 2], Zehua Peng[1, 2], Xuemu Li[1, 2], Bee Luan Khoo[3], Johnny C Ho[4, 5], Zhengbao Yang[1, 2, *]

[1]Department of Mechanical and Aerospace Engineering, Hong Kong University of Science and Technology, Clear Water Bay, Hong Kong, P. R. China

[2]Department of Mechanical Engineering, City University of Hong Kong, HKSAR, P. R. China

[3]Department of Biomedical Engineering, City University of Hong Kong, HKSAR, P. R. China

[4]Department of Materials Science and Engineering, City University of Hong Kong, HKSAR, P. R. China

[5]Department of Materials Science and Engineering, State Key Laboratory of Terahertz and Millimeter Waves, City University of Hong Kong, Kowloon, Hong Kong SAR, P. R. China

[6]Guangdong Provincial Key Laboratory of Functional Oxide Materials and Devices, Southern University of Science and Technology, Shenzhen 518055, Guangdong, P. R. China.

†Shiyuan Liu, Junchen Liao, Xin Huang, and Zhuomin Zhang contribute equally to this work.

*Corresponding author. E-mail: zbyang@ust.hk (Zhengbao Yang)

Supplementary Figures and Tables

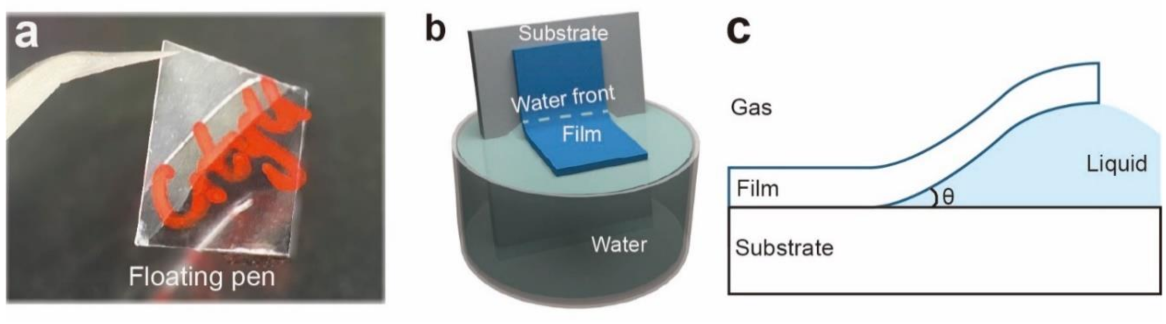

Fig. S1 Capillary transfer phenomena. (**a**) The "CityU" pattern, written in marker, is half on mica and half floating on water. (**b**) The schematic diagram of the capillary transfer. (**c**) The contact angle of the capillary transfer process

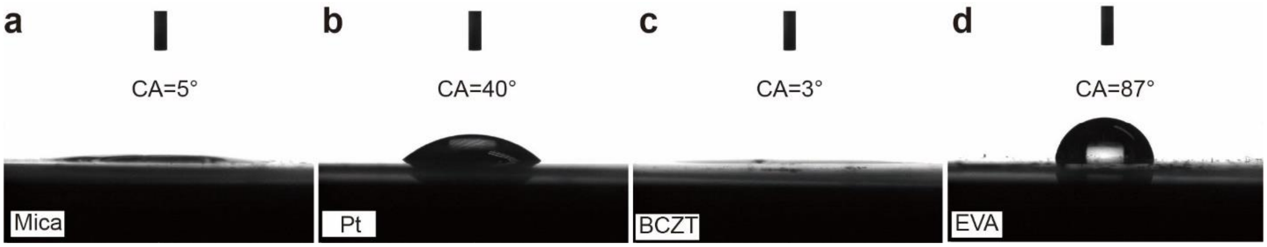

Fig. S2 The water contact angle of (**a**) mica, (**b**) Pt (on mica), (**c**) BCZT film (on mica), and (**d**) EVA (on mica)

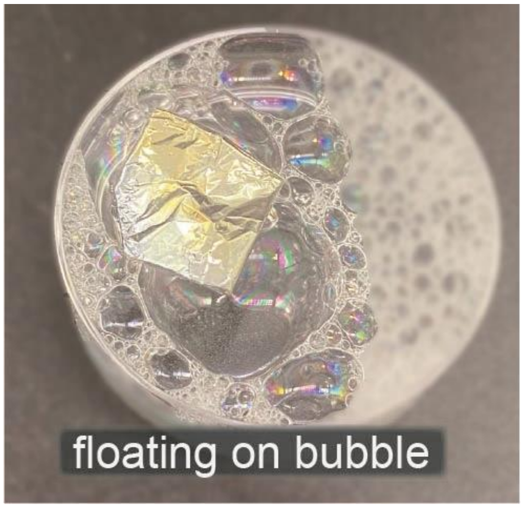

Fig. S3 The freestanding BCZT thin film with supporting layer EVA and bottom electrode Pt floats on water bubbles

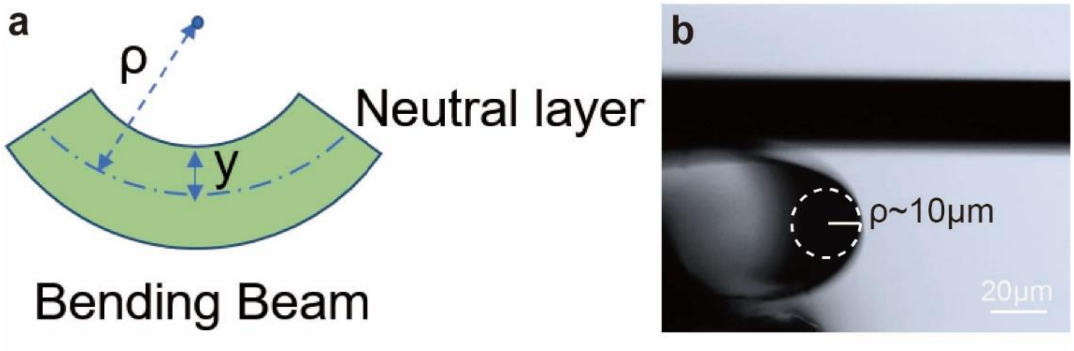

Fig. S4 The calculation of bending strain. (**a**) The parameter definition of the bending beam. The distance between the beam edge and the neutral layer is y, and the bending radius is ρ. The bending strain is calculated by $\boldsymbol{\varepsilon = y/\rho}$. (**b**) The bending BCZT thin film. The bending radius is estimated as 10 μm. The film thickness is about 0.4 μm. Thus the bending strain is ~2.0%

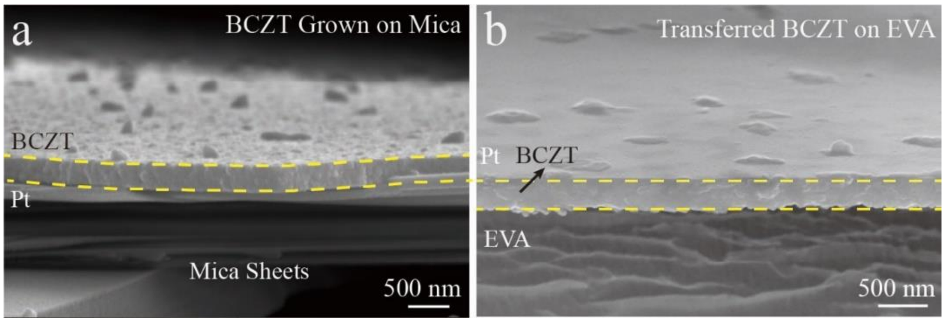

Fig. S5 SEM cross-sectional view of BCZT films. (**a**) The films grown on mica sheets. (**b**) The as-transferred BCZT film supported by EVA layer

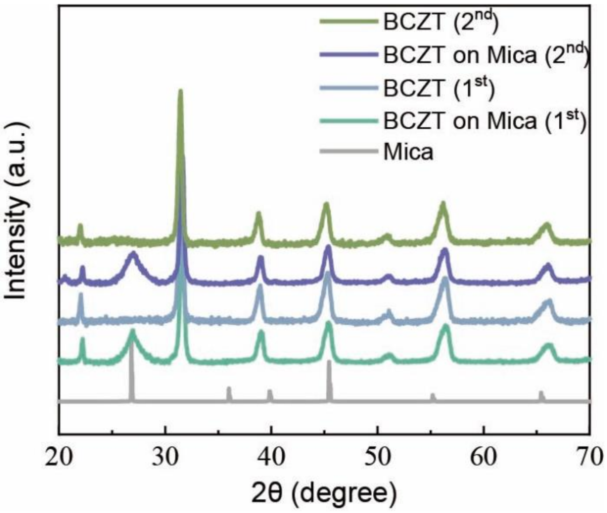

Fig. S6 XRD spectrum of mica, BCZT thin film grown on a single mica, and the transferred freestanding BCZT film (1st). To investigate the recyclability of the mica, another BCZT film is grown on the reused mica and transferred (2nd), which are characterized by XRD

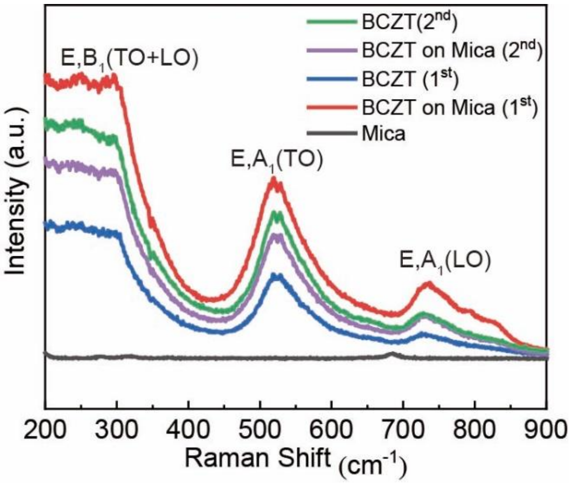

Fig. S7 Raman spectroscopy results of mica, BCZT thin film grown on a single mica for the first/second time, and the corresponding transferred BCZT thin film

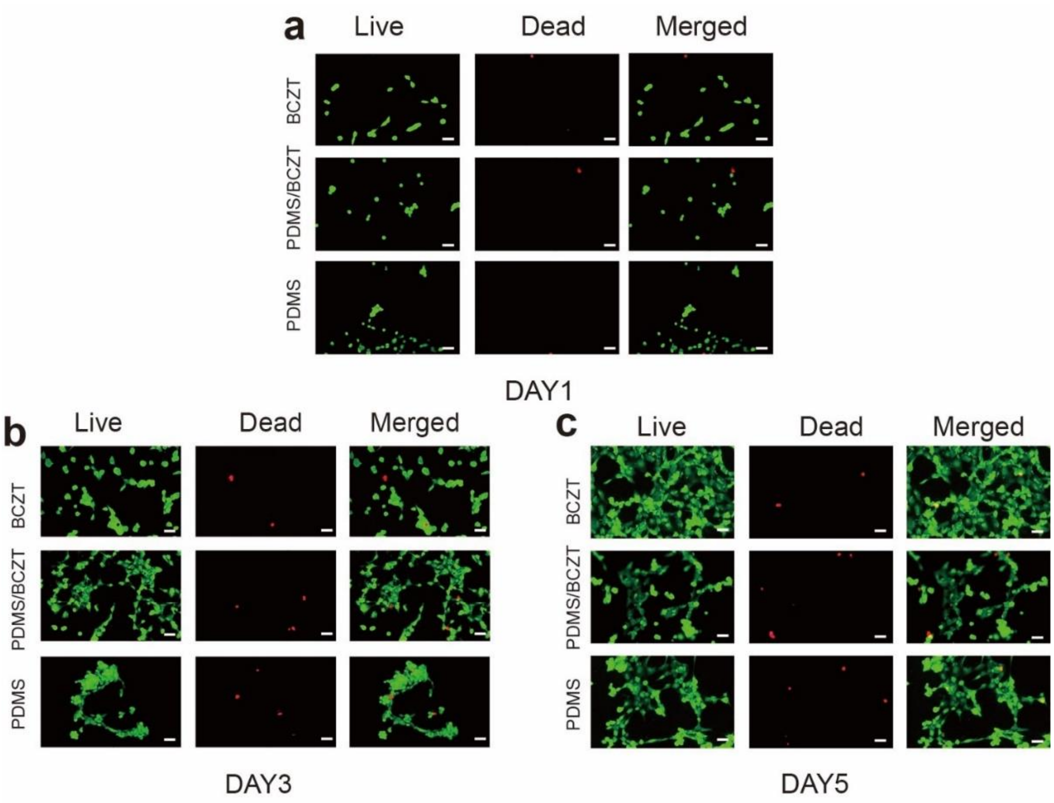

Fig. S8 The representative images of live and dead myofibroblasts on the surface of BCZT film, the BCZT film encapsulated by a 10 μm-thick PDMS layer, and a pure PDMS membrane after (**a**) 1 day, (**b**) 3 day, and (**c**) 5 day incubation

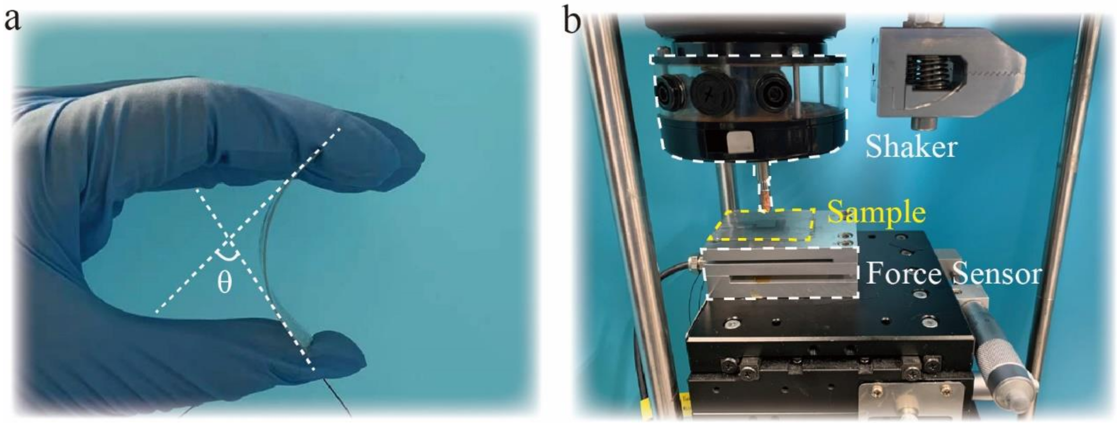

Fig. S9 Experiment setups of the piezoelectric performance tests. (**a**) The bending angle definition of the work. (**b**) The pressing experiment is conducted via a shaker under 40 Hz and the spontaneous pressing force is measured by an independent force sensor

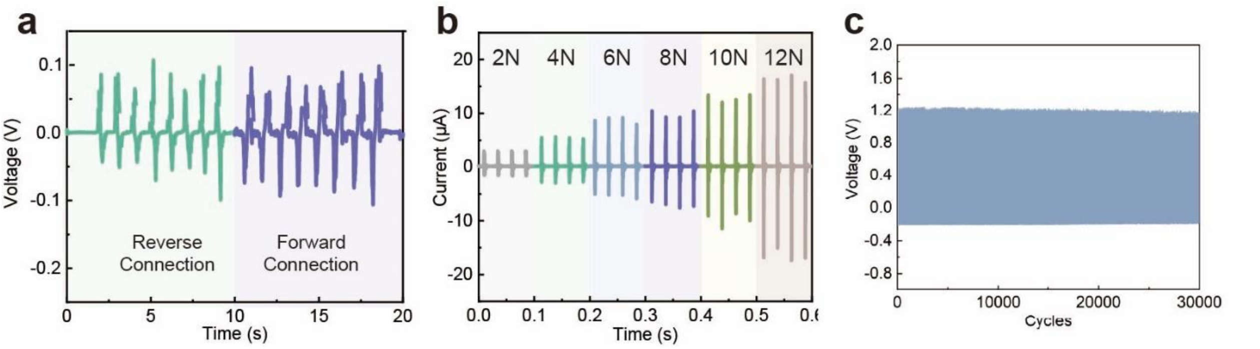

Fig. S10 Piezoelectric output of the energy harvester. (**a**) The opposite phase voltage output is obtained by connecting the energy harvester forward and reverse. (**b**) The short-circuit output of the PENG under different tapping forces. (**c**) The fatigue test shows the energy harvester remains stable under 30,000 times pressing

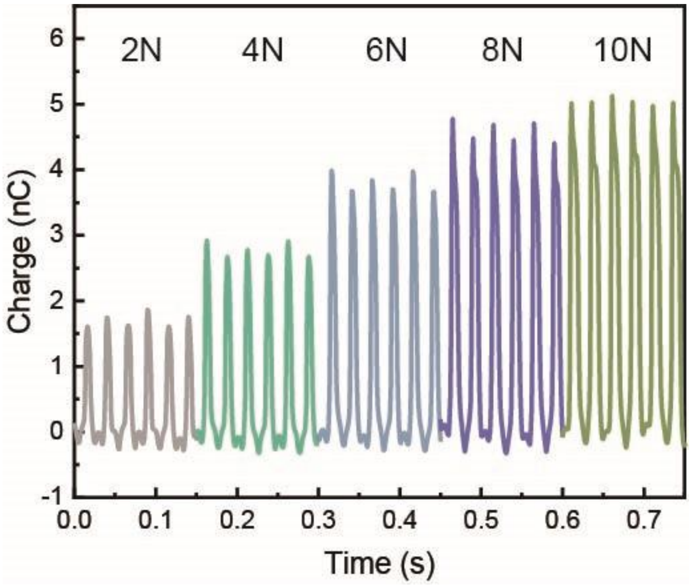

Fig. S11 Transfer charge of the FBEH under pressing stimuli

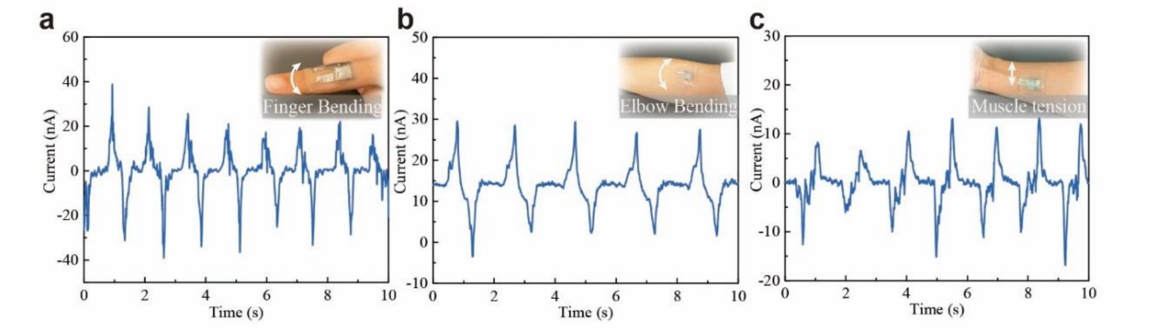

Fig. S 12 The sensing behavior of (**a**) finger bending, (**b**) elbow bending, and (**c**) the muscle tension and relaxation

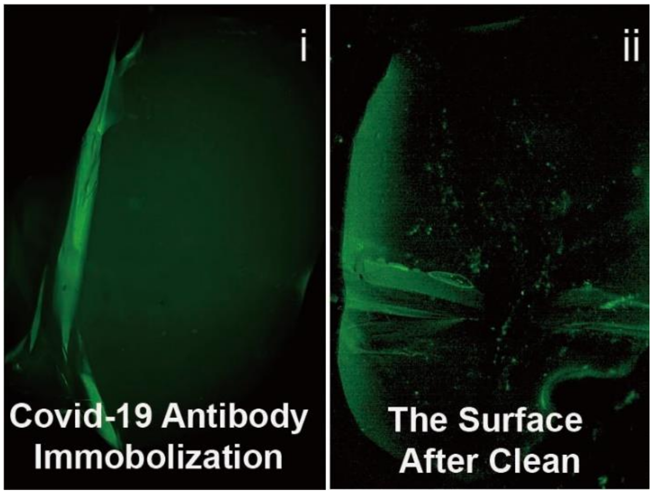

Fig. S13 The fluorescence microscopy images of fluorescently labeled covid-19 antibodies immobilized on the surface of grafted PDMS (i) before cleaning and (2) after cleaning

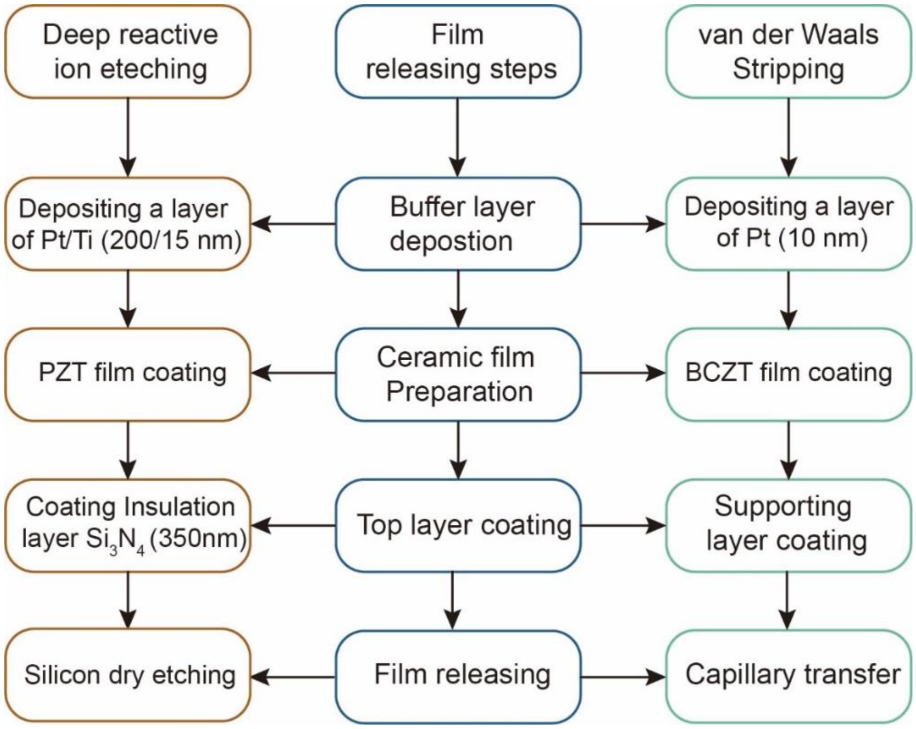

Fig. S14 Processing route of DRIE and vdW stripping

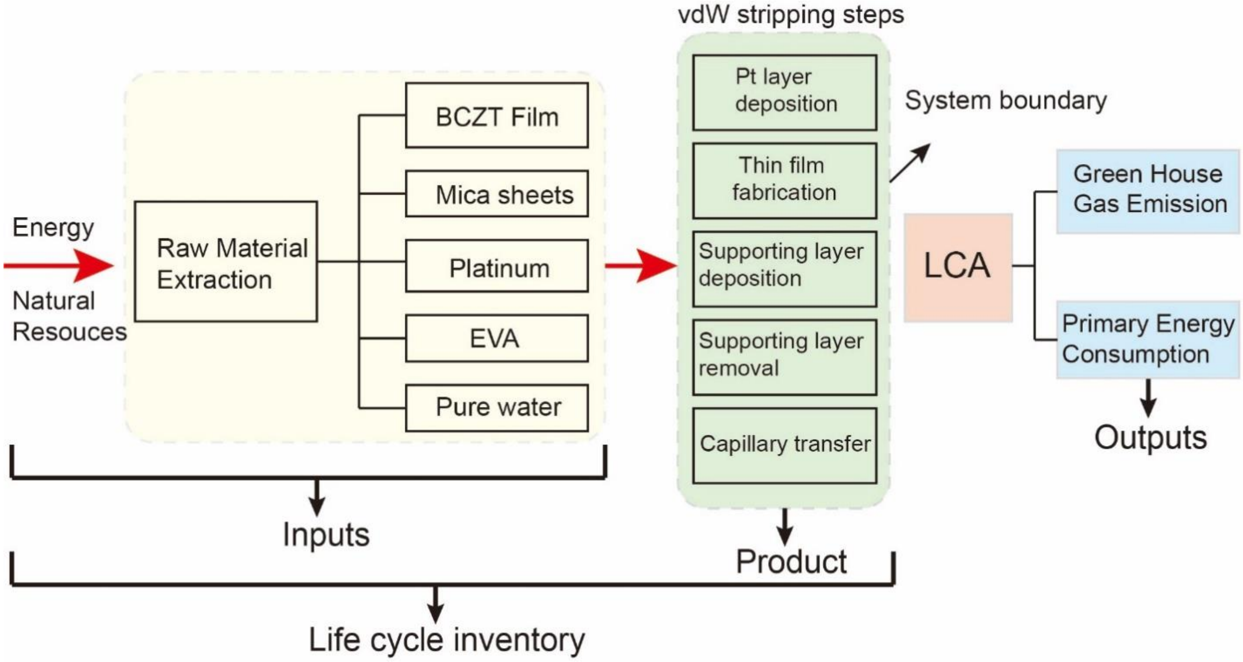

Fig. S15 System boundary and the life cycle inventory of the LCA

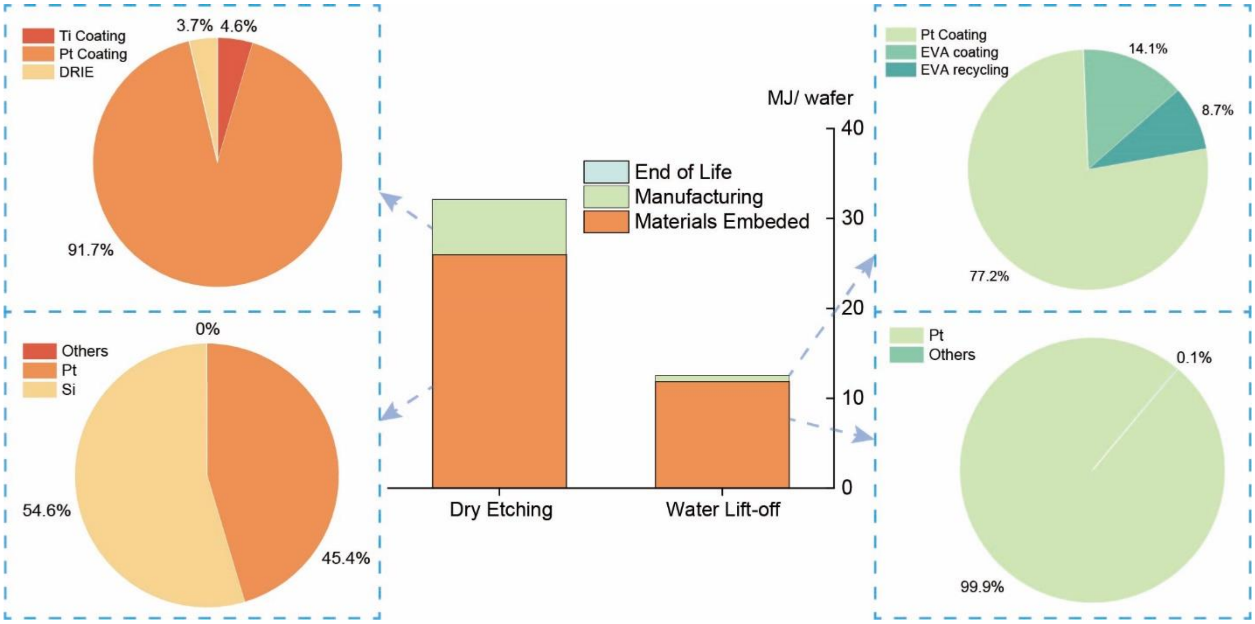

Fig. S16 Cumulative energy demand of the two processes

Table 1 Materials inventory of the substrate removal processes on a substrate of 2-inch diameter

Raw Materials	Mass (g)	Usage
Deep Reactive Ion Etching		
Titanium	1.66×10^{-4}	Electrode layer
Platinum	1.0411×10^{-2}	Electrode layer
Silicon wafer	5.378966	Substrate
Silicon nitride	2.692×10^{-3}	Insulating layer
Van Der Waals Stripping		
Platinum	1.04×10^{-3}	Buffer layer
Mica	1.349	Substrate
ethylene-vinyl acetate copolymer	2.23×10^{-2}	Supporting layer
Toluene	0.2	Organic solvent

Table 2 Energy consumption for removing film substrates of 2-inch diameter

Item	Power (W)	Time (s)	Electricity (MJ)
Deep Reactive Ion Etching			
Ti coating	1500	60	0.09
Platinum	1500	1200	1.8
Dry etching	600	120	0.072
Van Der Waals Stripping			
Pt coating	1500	120	0.18
EVA preparing	20	600	0.012
EVA baking	350	60	0.021

Video S1 The film bending test.
